# An Unusual Cause of Postaural Swelling: Kimura’s Disease

**Published:** 2017-07

**Authors:** Purodha Prasad, Swati Tandon, Vasun Batra, Ishwar Singh

**Affiliations:** 1 *Department of Otorhinolaryngology,* * Maulana Azad Medical College, New Delhi, India.*

**Keywords:** Angiolymphoid hyperplasia with eosinophilia, Eosinophilia, Kimura’s disease

## Abstract

**Introduction:**

Kimura’s disease (KD) is an allergic inflammatory disorder of unknown etiology endemic in Orientals. Kimura’s disease was first mentioned by Kimm and Szeto in China in 1937. Kimura’s disease is commonly encountered in Asia and is mostly reported in Japan, China, Singapore and Honkong. However, only a few cases have been reported in the Indian subcontinent.

**Case Report:**

A case of Kimura’s disease in a young male managed by surgery is reported in addition to a literature review.

**Conclusion:**

Diagnosis is made on the basis of histopathological analysis, clinical presentation, and laboratory investigations. Long term follow-up is required as Kimura’s disease is prone for recurrence.

## Introduction

Kimora et al observed multiple cases of Kimura’s disease in Japan and established its histopathological appearance in an article entitled “Unusual granulations combined with hyper plastic changes in lymphoid tissue”(1). Clinically, the lesion presents as a solitary or multiple subcutaneous nodules in the head and neck region. It usually has a benign progression but recurrences are common. It is commonly confused with Angiolymphoid hyperplasia (ALE) due to overlapping clinical and histopathological features. We report a case of KD in the postaural region that was initially thought to be ALE.

## Case Report 

**Fig 1:**
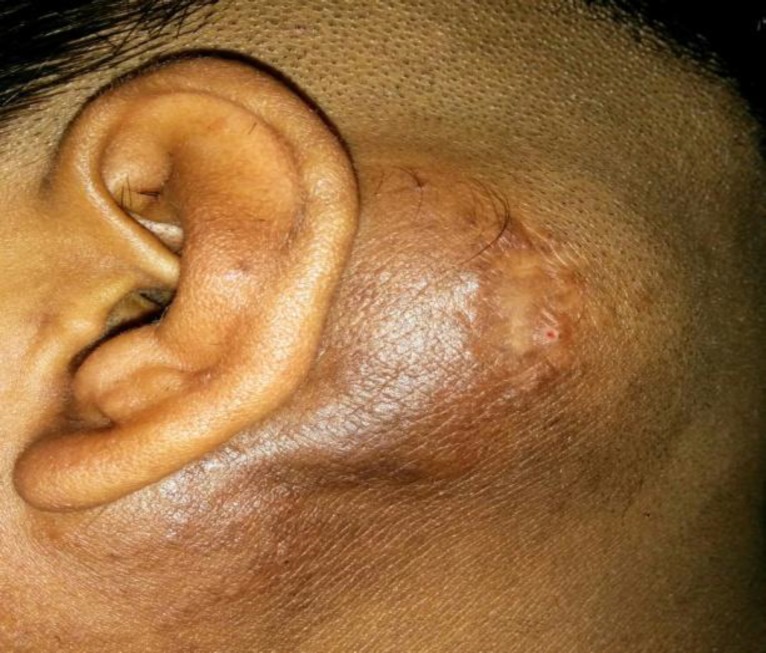
Clinical Photograph

An 18-year-old male presented with left postaural swelling, which was progressively increasing for 8 years. Occasionally, he complained of itching over the swelling. During clinical examination, the swelling was observed to be approximately 5 x 4 cm, solitary, non tender, firm, with ill defined margins (Fig.1). The overlying skin was thickened and erythematous. There was associated bilateral cervical lymphadenopathy. No similar swelling was found anywhere else in the body. There was no family or personal history of similar swellings.

**Fig 2:**
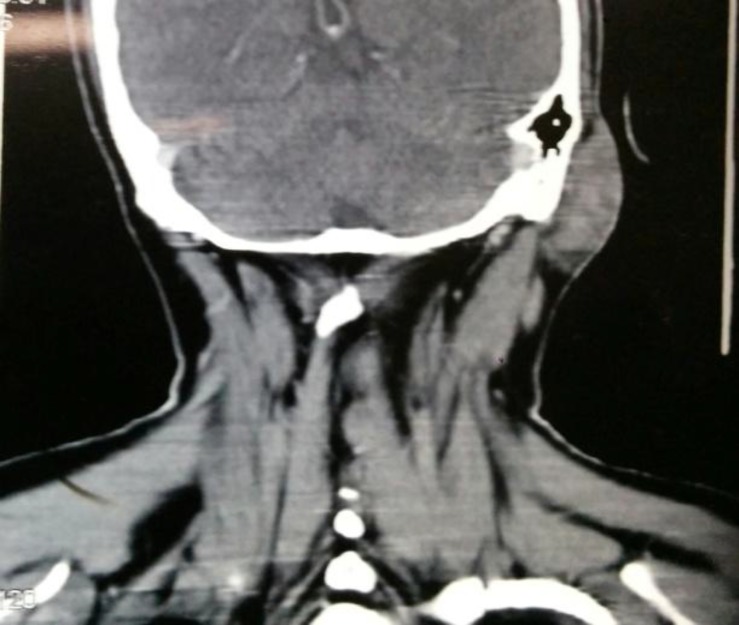
Ct Scan Showing Enhancing Lesion In The Subcutaneous Plane

During routine blood examination, the absolute eosinophil count was observed to be raised (2780). FNAC was inconclusive. Open biopsy, taken from the lesion under local anesthesia, was suggestive of angiolymphoid hyperplasia with eosinophilia. CT scan showed a homogeneously enhancing lesion in the subcutaneous plane (Fig.2). 

**Fig 3:**
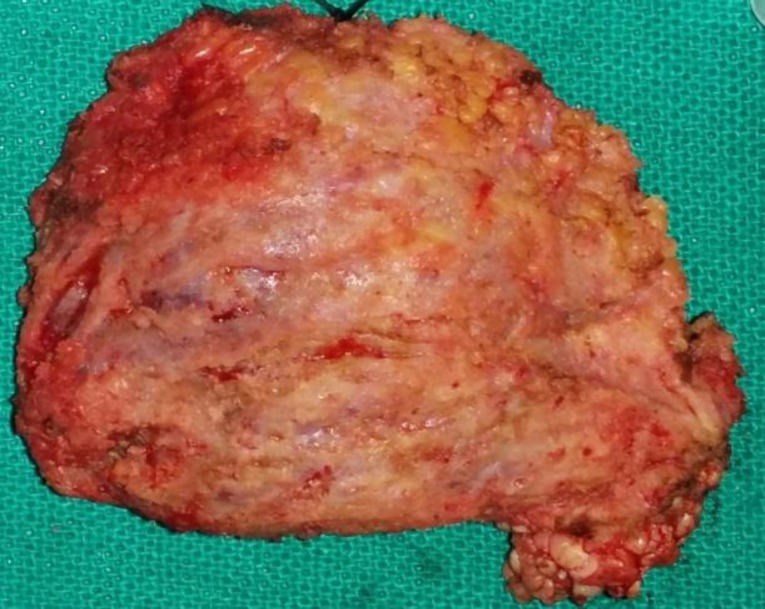
Postoperative Specimen

Meanwhile the patient was given oral steroids, anti-histaminic and diethylcarba- mazine but failed to achieve any reduction in the size of the swelling. Therefore, surgical excision of the swelling was performed under general anesthesia. Intraoperatively, the swelling was found in the subcutaneous tissue and was easily dissected out from the surrounding tissue in toto (Fig.3). 

**Fig 4:**
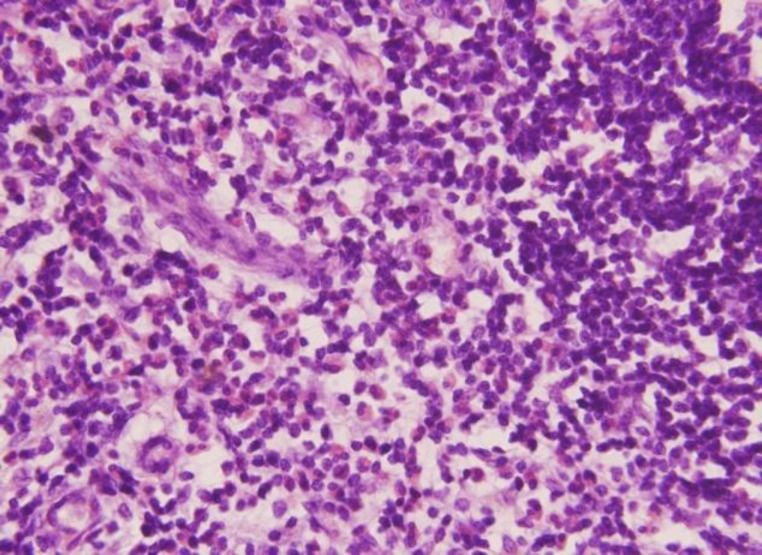
Histopathological Photograph

Postoperative histology showed diffuse fibrosis and infiltration by lymphoid tissue showing prominent reactive germinal centers with eosinophilia. The interstitial areas showed vascularization and infiltration by eosinophils consistent with Kimura’ disease (Fig.4). Currently, the patient is doing well with no recurrence observed during his 6-month follow up.

## Discussion

Kimura’s disease is a benign disease with recurrences as a common side effect. Kimura’s disease is most commonly confused with Angiolymphoid hyperplasia with eosinophilia. Other differential diagnoses include eosinophilic granuloma (2), Mikulicz’s disease, acute lymphocytic leukemia, Hodgkin’s disease, and Angioimmunoblastic lymphadeno- pathy (3). It usually affects young Asian males (M: F ratio is 3:1) (4).

Angiolymphoid hyperplasia with eosinophilia is an important differential diagnosis of Kimura’s disease. In both Angiolymphoid hyperplasia and Kimura’s disease, male preponderance, with involvement of the head and neck region, tendency to recur, and the vascular nature of the lesion with lymphoid and eosinophilic infiltrates has been encountered. However, ALE lesions are small and multiple and located mainly in the dermis. Whereas Kimura’s disease is a deep seated subcutaneous soft tissue swelling with cervical lymphadenopathy and involvement of salivary glands. However, the most important histological difference between the two is observed in blood vessels as the presence of “histiocytoid” and “epitheloid” blood vessels in ALHE, which are not observed in Kimura’s disease (5). In our case, the initially histopathology was reported as ALE but the postoperative report was consistent with KD.

Many theories have been proposed regarding the origin of Kimura’s disease, varying from allergic reaction to an alteration in immune regulation. Theories like persistent antigenic stimulation following arthropod bites and parasitic or candidal infection have been proposed. The current proposal is that T- cells have an important regulatory role in the development of eosinophilia and that T-cell derived cytokines contribute to different maturation stages for the production of eosinophils from the bone marrow. Therefore, Type 1 and Type 2 T helper cells (Th1 and Th2) are the principle factors resulting in an excess production of eosinophils and IgE which contribute to Kimura’s disease (6). Clinically, patients present with a swelling in the head and neck region mainly involving the subcutaneous tissue, major salivary glands and/or lymph nodes. The lesions appear painless, focal and present with single or multiple subcutaneous nodules. Pruritus is the most common complaint. In our case, the patient presented with postaural swelling, with pruritis being the only symptom. During routine hematological investigation, eosinophilia is almost always present. In our case, eosinophilia was seen and did not respond to oral steroids and antihelminthic drugs. During special investigations, elevated serum Immunoglobulin E level was observed. The only systemic manifestation is that of the renal system.

Treatment options include medical, surgical and ablation therapy. NSAIDS, corticosteroids, cryotherapy, intralesional chemotherapeutic agents (bleomycin, vinblastine and tacrolimus), surgical excision, radiotherapy, laser therapy are the various options for treatment (7). We administered steroids to our patient but neither the swelling reduced nor the eosinophilia responded. Surgical excision is considered the treatment of choice but carries recurrence rates of 33 - 50 %^7^. In our case, a surgical excision was performed with no recurrence observed during the 6-month follow up. We did not consider chemotherapeutic drugs and radiotherapy due to their side effects.

## Conclusion

KD is a benign disease. Otolaryngologists must be aware of this entity as it is one of the differential diagnoses of benign swelling in the head and neck region. Long term follow-up is required as it is prone to recurrence. 
